# Predicting Semantic Similarity Between Clinical Sentence Pairs Using Transformer Models: Evaluation and Representational Analysis

**DOI:** 10.2196/23099

**Published:** 2021-05-26

**Authors:** Mark Ormerod, Jesús Martínez del Rincón, Barry Devereux

**Affiliations:** 1 Institute of Electronics, Communications & Information Technology School of Electronics, Electrical Engineering and Computer Science Queen's University Belfast Belfast United Kingdom

**Keywords:** natural language processing, biomedical NLP, transformer models, representation learning, clinical text

## Abstract

**Background:**

Semantic textual similarity (STS) is a natural language processing (NLP) task that involves assigning a similarity score to 2 snippets of text based on their meaning. This task is particularly difficult in the domain of clinical text, which often features specialized language and the frequent use of abbreviations.

**Objective:**

We created an NLP system to predict similarity scores for sentence pairs as part of the Clinical Semantic Textual Similarity track in the 2019 n2c2/OHNLP Shared Task on Challenges in Natural Language Processing for Clinical Data. We subsequently sought to analyze the intermediary token vectors extracted from our models while processing a pair of clinical sentences to identify where and how representations of semantic similarity are built in transformer models.

**Methods:**

Given a clinical sentence pair, we take the average predicted similarity score across several independently fine-tuned transformers. In our model analysis we investigated the relationship between the final model’s loss and surface features of the sentence pairs and assessed the decodability and representational similarity of the token vectors generated by each model.

**Results:**

Our model achieved a correlation of 0.87 with the ground-truth similarity score, reaching 6th place out of 33 teams (with a first-place score of 0.90). In detailed qualitative and quantitative analyses of the model’s loss, we identified the system’s failure to correctly model semantic similarity when both sentence pairs contain details of medical prescriptions, as well as its general tendency to overpredict semantic similarity given significant token overlap. The token vector analysis revealed divergent representational strategies for predicting textual similarity between bidirectional encoder representations from transformers (BERT)–style models and XLNet. We also found that a large amount information relevant to predicting STS can be captured using a combination of a classification token and the cosine distance between sentence-pair representations in the first layer of a transformer model that did not produce the best predictions on the test set.

**Conclusions:**

We designed and trained a system that uses state-of-the-art NLP models to achieve very competitive results on a new clinical STS data set. As our approach uses no hand-crafted rules, it serves as a strong deep learning baseline for this task. Our key contribution is a detailed analysis of the model’s outputs and an investigation of the heuristic biases learned by transformer models. We suggest future improvements based on these findings. In our representational analysis we explore how different transformer models converge or diverge in their representation of semantic signals as the tokens of the sentences are augmented by successive layers. This analysis sheds light on how these “black box” models integrate semantic similarity information in intermediate layers, and points to new research directions in model distillation and sentence embedding extraction for applications in clinical NLP.

## Introduction

### Clinical Semantic Textual Similarity

Semantic textual similarity (STS) has long been an important task in natural language processing (NLP) research. Early work built document-level models for textual similarity that used an unsupervised approach, primarily for the purpose of indexing documents for search [1,2]. These models generally relied on the assumption that greater overlap in terms indicated greater interdocument similarity. This body of work was enriched by Lee et al [3] who also modeled similarity at the document level but elicited human semantic judgments of similarity to create a small data set of interest to NLP researchers and cognitive scientists. It was not until *SemEval-2012 Task 6* [4] that the first sentence-based STS data set was released, featuring 2000 training and 750 test sentence pairs that were rated by humans on a scale of 0-5 (from low to high similarity). Since then, there have been many new *SemEval* STS tasks, building on the initial task to encompass new domains of text [5] and cross-lingual similarity [6,7]. Researchers have used these models in a diverse set of applications such as discovering links between data sets [8] and identifying arguments in online discourse [9]. Recognizing both the potential of STS for processing eHealth records and the need for specialized data sets to account for clinical domain knowledge and handle the use of medical abbreviations, Rastegar-Mojarad et al [10] introduced a corpus of clinical sentence pairs that were assigned semantic similarity labels on a 0-5 scale by medical experts. This data set of 1068 annotated sentence pairs, as well as an expanded corpus of 174,629 unannotated sentence pairs, was released as MedSTS [11]. As with previous STS tasks, performance on this data set is measured by the Pearson correlation between the predicted labels and the ground-truth similarity scores. In general, the best systems in the *BioCreative/OHNLP Challenge STS task* used ensembles of traditional machine learning models and deep learning models [12], with the overall top-performing model achieving a correlation of 0.83 on the test set. The clinical STS task tackled in this paper, the *2019 n2c2/OHNLP Track on Clinical Semantic Textual Similarity* [13], uses an expansion of the *BioCreative/OHNLP Challenge STS task* data set.

### Transformer Models

In this work we train different types of transformer language models [14]. One of the types of transformer models that we train is bidirectional encoder representations from transformers (BERT) [15], which uses a masked language modeling task to train fully on bidirectional context without the decoder component of the original transformer architecture. Recently there has been much work in further training BERT on data from specialized domains, including biomedical text [16] and clinical documents [16-18]. We also further fine-tune these models on the task of STS. The last type of transformer model that we fine-tune is XLNet [19], which performs autoregressive language modeling while also capturing bidirectional context by sampling different possible word orders.

### Interpreting Deep Neural Networks

After we train our models, we explore the representations that they build of clinical semantic similarity to identify any systematic biases or heuristics they may have learned that we can then work toward addressing to improve future clinical STS transformer architectures. There is a substantial literature that uncovers the kind of linguistic representations deep neural networks learn by experimentally perturbing the model’s input and carefully analyzing the failure cases [20-22]. Another approach uses “decoding” to try to predict task-relevant information from intermediate representations generated from the model [23-25]. Recently there has been further work on interpreting the representations in deep neural models using attention weights [26,27]. While this approach is intuitive, there is still an ongoing debate about the extent to which the attention mechanism can be used to interpret a model’s decision-making process [28,29]. As such, we focus our layer-wise analysis on our models’ hidden token vectors [24]. Other relevant work on layer-wise analyses of BERT representations include [30] and [31].

One method we use to analyze the representational geometry of our models is representational similarity analysis (RSA) [32], which compares models that represent stimuli using vectors with different numbers of dimensions by measuring the correlation of second-order dissimilarity matrices with each other (ie, how dissimilar each pair of sentences is to each other pair by some metric). RSA has been used recently to analyze linguistic properties of deep learning models [33,34]. We use basic RSA to correlate various representations that we extract from each layer of our fine-tuned models with a matrix that corresponds to the ground-truth dissimilarity patterns found in the test set. This allows us to measure the strength of a clinical semantic signal through the layers of our networks and compare this signal across both models and choices of representation. We also employ a version of RSA that involves reweighting and linearly recombining the representational dissimilarity matrices (RDMs) [35] to build a representational model that best explains the ground-truth dissimilarity patterns in the test set. To our knowledge, this is the first use of this framework to explore the representational space of a deep neural language model.

### Contributions

This work presents the following contributions:

- A transformer ensemble that achieves very competitive results on a new clinical STS task (with predictions producing a correlation of 0.87 with ground-truth similarity scores compared with the state-of-the-art correlation of 0.9), serving as a very strong deep learning baseline for this task.
- An extensive qualitative analysis of the transformer ensemble’s error cases in the task of clinical semantic similarity that highlights the inability of popular transformer models to capture fine-grained differences between medicinal sentence pairs, despite being trained on clinical or biomedical text.
- A quantitative error analysis framework for STS that reveals the shallow heuristics that transformer models learn to rely on for this task.
- The application of linear decoding and RSA to measure the semantic similarity signal in intermediate token representations of 5 popular transformer models, showing convergent and divergent representational strategies that reflect the models’ performance on this task.
- The first application (to the authors’ knowledge) of a reweighted and recombined version of RSA to neural language models, indicating that better representations of sentence pairs may be synthesized by combining 2 layers from a relatively poorly performing biomedical transformer with a simple textual feature signal, and suggesting new directions for research in sentence embedding extraction.

## Methods

### Data

The training data for this task were made up of 1642 sentence pairs and their associated similarity scores and the test set was made up of 412 sentence pairs. The similarity scores are floats on a scale of 0 to 5, ranging from no similarity to semantically identical. The annotations were performed by 2 medical experts (Donna Ihrke and Gang Liu [13]). The task is evaluated by the Pearson correlation between the predictions of a model and the ground-truth similarity scores.

### Models

**Figure 1:**
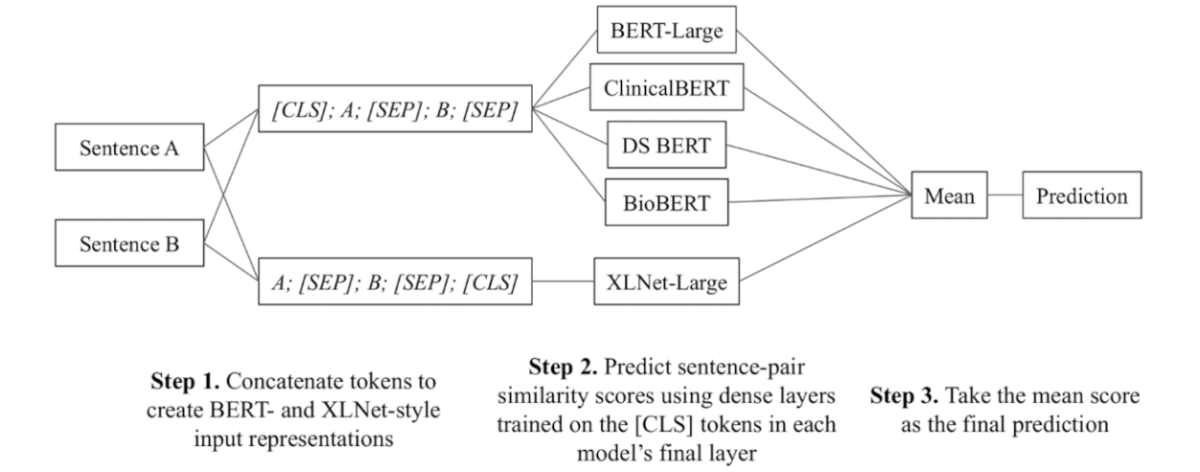
Our system architecture for predicting the semantic textual similarity between two sentences using an ensemble of five Transformer models.

We fine-tuned 5 transformer [14] models. These include BERT-Large [15], 3 variants of BERT that were fine-tuned on text from the clinical domain, and XLNet-Large [19]. The 3 BERT variants were BioBERT [16], ClinicalBERT [17,18], and Discharge Summary BERT (DS BERT) [17,18]. We also created a *mean_score* model by taking the average prediction of the 5 transformer models. A linear layer was added on top of the pooled output for each model to perform the regression. The input for the BERT models was *[CLS] + A + [SEP] + B + [SEP]*, where *[CLS]* is the classification token, *A* and *B* are the 2 text snippets, and *[SEP]* is the separator token. The input for XLNet was *A + [SEP] + B + [SEP] + [CLS]*. We set the maximum sequence length for each model to 128. As we add 3 additional tokens to the input, any sentence pairs with over 125 tokens in total were shortened. This affected 5 sentence pairs, all of which were in the training set (with an average of 7.6 removed tokens). Each model was trained over 23 epochs using a batch size of 32. These models were trained using the PyTorch-Transformers library [36]. Our system architecture is depicted in Figure 1. We submitted the predictions of 3 models for evaluation on the n2c2 2019 Track 1 task: those from ClinicalBERT, XLNet, and the mean_score model.

## Results

### Overview

Our best performing model, the mean_score ensemble, achieved a correlation of 0.87, reaching 6th place out of 33 teams in the n2c2 2019 Track 1 task. The best model on the task achieved a correlation of 0.9 [37]. Our results are presented in Table 1. The correlation between the predictions of each of 5 transformer models with all others is presented in Table 2. While the 3 models that have been fine-tuned with biomedical or clinical text (BioBERT, ClinicalBERT, and DS BERT) are more correlated with each other than with both XLNet and BERT, the predictions of all models generally correlate strongly with each other.

**Table 1:** Pearson correlation between the ground-truth labels and the predicted labels for each model.

Model	BERT	BioBERT	ClinicalBERT	DS BERT	XLNet	Mean score
Correlation	0.817	0.855	0.854	0.867	0.837	0.870

**Table 2:** Correlation between the predictions of each transformer model on the test set.

Model	BERT	BioBERT	ClinicalBERT	DS BERT	XLNet
BERT	1	0.92	0.92	0.92	0.91
BioBERT	0.92	1	0.95	0.96	0.92
ClinicalBERT	0.92	0.95	1	0.96	0.92
DS BERT	0.92	0.96	0.96	1	0.93
XLNet	0.91	0.92	0.92	0.93	1

### Error Analysis

#### Error Cases Investigation

Rather than only evaluating our transformer ensemble by the correlation between its predictions and the ground-truth similarity scores, we carried out an extensive investigation into the error cases of this ensemble to shed light on any trends in the biases and heuristics that the component models may have learned from the training data. In this endeavor we carried out both qualitative and quantitative error analyses. Both analyses use a measure of loss that is calculated as the squared error between the models’ prediction and the ground-truth similarity score.

#### Qualitative Analysis

**Figure 2:**
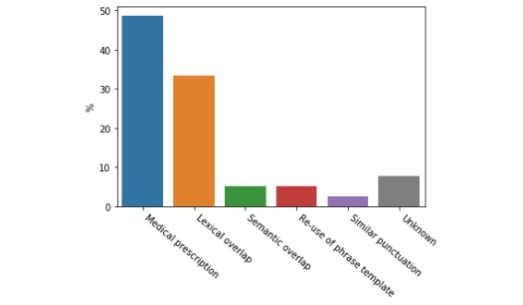
Common categories of error for cases when the model over-predicts similarity as identified by manual analysis of the 100 worst predictions.

**Figure 3:**
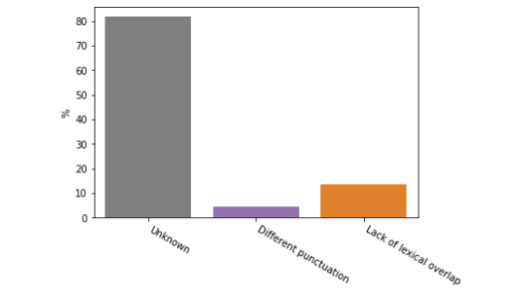
Common categories of error for cases when the model under-predicts similarity as identified by manual analysis of the 100 worst predictions.

We first carried out a qualitative analysis by grouping the sentence pairs that were most difficult to predict for the transformer ensemble by the primary lexical, syntactic, or semantic feature that we consider to be most salient and distinguishing. By identifying common error clusters, we can better understand our models’ biases and attempt to mitigate these issues in future iterations of the clinical STS system. A list of these error categories as well as example sentences can be found in Table 3. We took 100 sentence pairs from the test data set with the highest loss and manually analyzed them to find possible explanations for incorrect predictions. The main categories that were identified are shown in Figures 2 and 3. We divided the errors into 2 cases: those where the transformer ensemble overpredicted sentence similarity with respect to the ground truth (Figure 2, which includes 77 sentence pairs) and those where the models underpredicted sentence similarity (Figure 3, which includes 23 sentence pairs).

**Table 3:** Example sentence pairs and error type (ie, whether the transformer ensemble overpredicted or underpredicted semantic similarity with respect to the ground truth) for each error category selected for the qualitative analysis.

Error type	Category	Example sentence pair	Notes
Overprediction	Medical prescription	(1) Ibuprofen [MOTRIN] 400 mg tablet 1 tablet by mouth every 4 hours as needed. (2) Gabapentin [NEURONTIN] 300 mg capsule 1 capsule by mouth every bedtime.	
Overprediction	Lexical overlap	(1) Patient to call to schedule additional treatment sessions as needed otherwise patient dismissed from therapy. (2) Patient tolerated session without adverse reactions to therapy.	
Overprediction	Semantic overlap	(1) The client verbalized understanding and consented to the plan of care. (2) The patient consented to the possibility of blood transfusion.	Some semantic overlap despite low ground-truth similarity score of 0
Overprediction	Reuse of phrase template	(1) male who presents for evaluation of Knee Pain (right). (2) female who presents for evaluation of Ear Infection/ Ear Pain.	Common phrase structures often feature lexical overlap, as well as strong syntactic similarity
Overprediction	Similar punctuation	(1) “Left upper extremity: Inspection, palpation examined and normal.” (2) “Abdomen: Liver and spleen, bowel sounds examined and normal.”	Note quotation marks within original text
Overprediction	Unknown	(1) “Mental: Alert and oriented to person, place and time.” (2) She demonstrated understanding and agreed to proceed as noted.	The ensemble predicted a score of 2.55/5 for this example sentence pair
Underprediction	Unknown	(1) He denies any shortness of breath or difficulty breathing. (2) Patient denies any chest pain or shortness of breath.	
Underprediction	Different punctuation	(1) “Thank you for choosing the Name, M.D.. care team for your health care needs!” (2) Thank you for choosing Location for your health care and wellness needs.	
Underprediction	Lack of lexical overlap	(1) The above has been discussed and reviewed in detail with the patient. (2) The family was advised that the content of this interview will be shared with the health care team.	Semantic similarity with little lexical overlap

#### Quantitative Analysis

To complement our qualitative analysis, we developed a simple STS quantitative analysis framework that allows us to investigate the relationship between surface features of the sentence pairs and our model’s performance. This involves measuring the correlation between model loss and various features of the sentence pairs. In addition to providing the results for all labels, we present correlations (measured using Spearman rho) between the loss and pair features for each similarity score in the test set. The results are shown in Table 4. Below is an explanation of each sentence-pair feature that we investigated:

- Average sentence length: The total amount of tokens across the 2 sentences.
- Scaled total token frequency: The number of times each token in the sentence pair appears in the training set divided by the average sentence length, calculated after we removed stop words.
- Scaled unseen tokens per pair: The number of tokens in the sentence pair that do not appear in the training corpus, divided by the average sentence length.
- Scaled difference in token frequency: The difference between the training corpus token frequency across the 2 sentences, divided by the average sentence length, calculated after we removed stop words.
- Jaccard distance: The distance between the token sets of 2 sentences in a pair measured as

1 – (|A ∩ B|)/(|A ∪ B|)

**Table 4:** Correlation (Spearman rho) between the model’s loss (mean score) per sentence pair and various sentence-pair features.

Label^a^	Average sentence length	Scaled total token frequency	Scaled unseen tokens per pair	Scaled difference in token frequency	Jaccard distance
All	−0.132	0.142	0.020	0.074	−0.025
0.0	−0.310	0.391	−0.263	0.219	−0.554 (<.001)^b^
0.5	0.102	−0.114	−0.249	−0.010	−0.202
1.0	0.067	−0.043	0.047	−0.033	−0.074
1.5	0.004	−0.151	0.033	−0.281	−0.153
2.0	0.118	0.441	0.012	0.354	−0.338
2.5	−0.018	0.014	−0.238	0.070	0.109
3.0	−0.453	0.432	−0.098	−0.026	0.119
3.5	−0.440	−0.051	0.257	−0.046	0.587
4.0	−0.088	0.138	0.268	0.052	0.171
4.5	−0.181	−0.266	−0.221	0.033	0.468
5.0	−0.040	0.789 (.042)	−0.242	0.590	0.596

^a^Labels are ground-truth similarity scores.

^b^Significant *P* value is reported in parenthesis after Bonferroni correction.

### Layer-wise Token Representation Decoding

Given the difficulty of analyzing how these models build representations of clinical STS by looking at their loss alone, we next performed a layer-wise decoding analysis by training linear regression models to predict between-sentence semantic similarity given representations from each transformer across different layers of the model. By decoding the semantic signal in the intermediate layers of each model, we can uncover the mechanisms that transformer models use to predict clinical semantic similarity. We can then investigate whether any representational strategies correspond to better performance on this task, shedding light on why certain constituent models of the transformer ensemble perform worse, and potentially indicating directions for sentence-pair embedding extraction for STS. In the case of 12-layer models we used each layer and in the case of the larger 24-layer models, we used every other layer. This allows for direct comparison of representations by relative depth through the network.

We chose a variety of representations to decode. As we have many tokens per sentence pair, there are many different possible ways to map this list of vectors to a fixed-length representation. We aimed to choose representations that can reveal potential strategies and heuristics that our models use to predict semantic similarity. In doing so, we may also reveal how different types of models (ie, those trained on clinical versus general domain text, or those with BERT/XLNet-style architectures) diverge or converge in their representational transformation strategies. The chosen representations were

- [CLS]: The token vector corresponding to the classification token input.
- avg_reps_concat: Concatenation of the mean-pooled token vector representations of sentences A and B.
- max_reps_concat: Concatenation of max-pooled token vectors within sentences A and B.
- sent_avg_difference: The absolute difference in average token vector representations in sentences A and B.
- sent_max_difference: The absolute difference in max-pooled token vector representations in sentences A and B.
- sent_a_avg_max_concat: Concatenation of mean- and max-pooled token vectors from sentence A.
- sent_b_avg_max_concat: Concatenation of mean- and max-pooled token vectors from sentence B.

**Figure 4:**
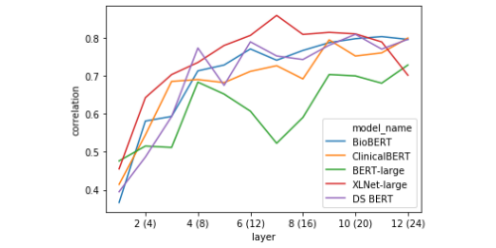
Pearson correlation between linear regression models’ predictions of a sentence pair’s semantic similarity and the ground-truth score (10-fold cross-validated on test-set) using [CLS] token pair representations.

**Figure 5:**
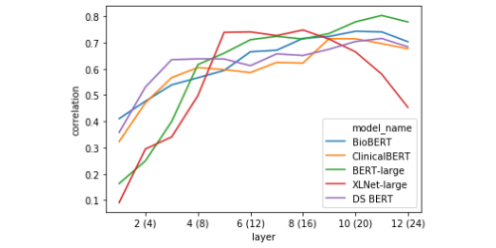
Pearson correlation between linear regression models’ predictions of a sentence pair’s semantic similarity and the ground-truth score (10-fold cross-validated on test-set) using the absolute difference between each sentence’s mean-pooled token vector.

The linear regression models were evaluated using 10-fold cross-validation. Table 5 shows the overall best representations for decoding similarity score. Figures 4 and 5 feature layer-wise correlation plots for representations based on the classification token vector (Figure 4) and the absolute difference between the average token vectors in each sentence (Figure 5).

**Table 5:** The overall top decoding scores ranked in descending order. All the top-performing representations were extracted from XLNet and are mostly made up of the concatenation of the max-/mean-pooled token representations in the 2 sentences that were extracted from middle-late layers.

Model	Representation	Layer	Correlation
XLNet-large	max_reps_concat	18	0.9
XLNet-large	sent_a_avg_max_concat	18	0.89
XLNet-large	avg_reps_concat	18	0.88
XLNet-large	max_reps_concat	20	0.88
XLNet-large	avg_reps_concat	16	0.88
XLNet-large	avg_reps_concat	20	0.88
XLNet-large	sent_b_avg_max_concat	18	0.87
XLNet-large	sent_b_avg_max_concat	14	0.87
XLNet-large	max_reps_concat	14	0.87
XLNet-large	max_reps_concat	16	0.87

### Representational Similarity Analysis

#### Overview

To find which representations learned by our models best explain the representational geometry of the semantic similarity task, we carried out 2 types of investigations within the framework of RSA. We use RSA to complement our layer-wise linear probing analysis, as it can reveal second-order representational patterns across many samples, while the layer-wise probing analysis relies on identifying particular dimensions of the representational space that predict semantic similarity. By taking these methods together, we can reach more robust conclusions about how transformer models build representations of semantic similarity and use this information to understand the performance of these models and identify how we can improve them. The data RDMs that we compared with the ground-truth RDM were extracted from each layer of each of the 5 transformer models, for each of the pair representations defined in the previous decoding analysis as well as 3 additional potential explanatory representations:

- avg_representation: The average across all token vectors.
- avg_sent_cosine_dist: The cosine distance between the mean-pooled token vector representations in sentences A and B.
- max_sent_cosine_dist: The cosine distance between the max-pooled token vector representations in sentences A and B.

#### Basic RSA

**Figure 6:**
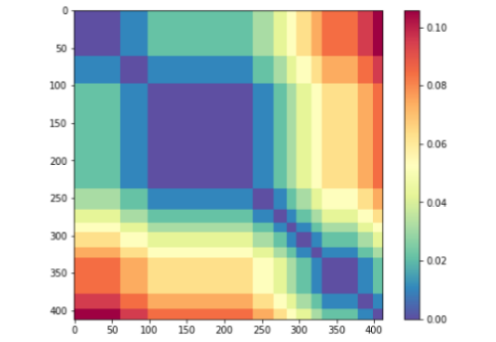
Model representational dissimilarity matrix for 412 test sentence pairs measured by distance between ground-truth semantic similarity scores. The dimensions of the dissimilarity matrix are sorted by each sentence-pair’s ground-truth semantic similarity score.

In our first RSA experiment, we performed a basic analysis in which we measure the Spearman correlation between a model RDM (calculated using the distance between all the samples in the test set measured by their ground-truth similarity score) and various representations elicited from our transformer models. Using the 412 test sentence pairs we produced the 412 × 412 matrix shown in Figure 6.

#### Reweighted and Recombined RSA

**Figure 7:**
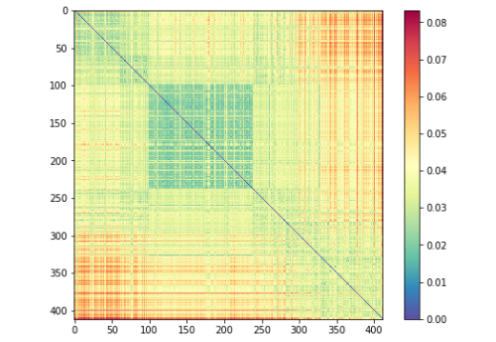
The final best-fitting re-weighted and linearly re-combined explanatory model found using NNLS and representations from BioBERT, achieving a correlation of 0.54 with the ground-truth model. The dimensions of the dissimilarity matrix are sorted by each sentence-pair’s ground-truth semantic similarity score.

We then found a combination of representations from all layers of each of the separate 5 transformer models and an RDM made up of text features (detailed in the “Quantitative Analysis” section) that best explains the ground-truth model when linearly recombined. Each explanatory RDM in a given trial had an associated weight that altogether summed to 1. These weights were found using a non-negative least squares (NNLS) solver using 10-fold cross-validation. This analysis revealed that the best performing explanation model was BioBERT. The final BioBERT-reweighted explanatory RDM is shown in Figure 7.

#### Layer-wise Reweighted RSA

**Figure 8:**
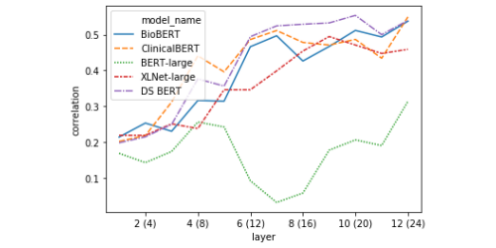
Correlation between the ground-truth model RDM and explanatory RDMs constructed from [CLS] token pair representations.

**Figure 9:**
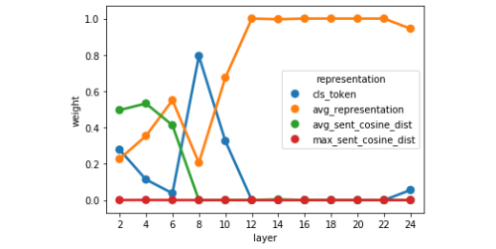
Weights associated with sentence-pair representations of BERT-Large found using NNLS to minimise the distance between a linearly re-combined set of RDMs and the ground-truth model RDM for each layer.

In the final part of our reweighted RSA, we revisited the representations of BERT-Large to investigate why the classification token suddenly becomes less representative of the ground-truth similarity score around layers 12-16 as measured by linear regression probing (Figure 4) and RSA correlation (Figure 8). We reran the NNLS solver for the BERT-Large representations (using 10-fold cross-validation) but this time we excluded the text features RDM and used token vectors from only 1 layer at a time. We performed this analysis for the even layers, from layers 2 to 24 (as we had previously extracted every other layer of the 24-layer models to directly compare representations with 12-layer models based on relative depth through the network), and retrieved the values used to reweight the RDM for each layer. The plot of weights associated with each representation can be seen in Figure 9.

## Discussion

### Principal Results

#### Qualitative Error Analysis

In the case of sentence pairs that caused our ensemble to overpredict semantic similarity (Figure 2), the most obvious problem with our ensemble was its failure to model the semantic similarity of 2 sentences which contain details of medical prescriptions. This is likely because our models do not have the advanced level of domain knowledge necessary to correctly model this problem. As these sentences are usually very similar (apart from the name of a drug and dosage), the models overpredict similarity. The second biggest issue when overpredicting similarity is when there is a lexical overlap without semantic overlap. This suggests that our models over-rely on surface features such as token overlap. In most cases when our model underpredicts similarity, there is no obvious possible explanation. However, in the interpretable samples the issue was usually that synonyms were used, again suggesting an over-reliance on lexical overlap, and potentially motivating a concept normalization preprocessing step. In any case, the qualitative approach to analysis error is relatively limited for interpreting the instances of underprediction of semantic similarity for this ensemble. This limitation is mitigated by the fact that overpredictions made up the majority of the largest errors (77 out of 100). By taking both the cases of underprediction and overprediction together, it is clear that simple heuristics, such as predicting similarity given lexical overlap, are prominent within the transformer ensemble, and that these transformer models still lack the ability to produce the extremely fine-grained clinical semantic representations that are required to implicitly calculate semantic distances between medical concepts (eg, particular drugs) given a relatively small task set. Any future work would have to address these issues; for example, by augmenting the data using a concept normalization preprocessing step, or by enriching the ensemble’s domain knowledge by incorporating a clinical terms resource.

#### Quantitative Error Analysis

Overall, Table 4 shows a weak negative correlation between the average sentence length and loss. This relationship is relatively strong for entirely dissimilar sentence pairs and moderately similar sentence pairs and may be explained by the fact that longer sentences provide more contextual information that can be used to decide whether 2 sentences are semantically similar. Another trend is for the loss to increase with the scaled total token frequency (ie, how often the words in the pair appear in the training corpus), particularly in the case where the 2 sentences are semantically identical. This relationship is difficult to interpret, but additional analysis could investigate the extent to which the loss can be explained using the relative frequency of the words given a more general corpus (such as Wikipedia), to separate the effect of clinical term frequency.

We also see that Jaccard distance is negatively correlated with loss for sentence pairs that are less semantically similar and positively correlated with loss for pairs that are more semantically similar. One possible explanation for this observation is that our deep transformer models have learned an appropriate strategy of predicting low similarity scores given token overlap for the extreme case when sentence pairs are dissimilar and have little overlap. However, the model seems unable to apply such a shallow heuristic in cases where sentence pairs are very semantically similar. Further analysis showed Jaccard distance to be very significantly negatively correlated with the ground-truth label (*P*<.001), which may indicate that a deep ensemble model could benefit from the presence of traditional machine learning models that are trained on simple features of the text such as relative overlap between tokens.

The quantitative analysis approach has both verified the existence of overall heuristics that use surface features of the sentence pairs to predict semantic similarity as noted in the previous qualitative analysis and allowed to us examine these trends as they occur within certain ranges of semantic similarity scores. This approach to quantitative analysis of STS errors has thus produced a richer view of these biases, while still suggesting that these deep transformer models use a set of relatively shallow strategies for this task.

#### Layer-wise Token Representation Decoding

The first striking pattern to note in Figure 4 is that the BERT models tend to drop in performance on the CLS token task in the middle of the network, thereafter reaching their apexes (in the extreme case this is amplified in BERT-Large), whereas XLNet tends to steadily increase to its highest point before dropping off over the rest of the network. This indicates that in BERT-style models, the [CLS] token does not serve as the primary representation of semantic similarity in the middle layers. Second, the correlation between linear model predictions and ground-truth scores held-out folds almost always monotonically increases for the difference between average sentence representations for all BERT-style models (Figure 5). This contrasts with the performance on the XLNet sent_avg_diff representation, which caps half-way through the network, then drops off steadily beginning a few layers later. It appears that XLNet builds a good representation based on the mean-pooled token representations, but that this information is integrated in the middle of processing and subsequently discarded around layer 18.

All the top 10 best decoding scores across all representations were extracted from XLNet (Table 5). Overall, XLNet did best using the max_reps_concat, reaching a correlation of 0.9 in layer 18, which represents a 7.5% increase in that model’s initial performance on the test set. This demonstrates that given the initial representations of a large deep model, it may be possible to increase its performance very inexpensively and massively on small amounts of held-out data using a simple linear model and the correct choice of representation.

It is clear from the linear decoding experiment that the representational strategies of the transformers fine-tuned with biomedical or clinical documents tend to align, with each model gradually building better representations of STS over the course of their layers in an almost always monotonic fashion, in both the [CLS] token and the absolute difference between mean-pooled sentence representations. This is in contrast to the relatively erratic differences between decodability over layers seen with BERT-Large and XLNet, where decodability will rapidly gain or fall over the course of 1-2 layers, especially when looking at the distance between mean-pooled sentence vectors representation. This result suggests that models with more clinical domain knowledge (and better performance on this task) learn to build robust representations of clinical semantic similarity (ie, not only using the [CLS] token or the distance between mean-pooled vectors) and that this information is gradually recovered in a steady, step-wise manner.

#### Representational Similarity Analysis

##### Basic RSA

**Figure 10:**
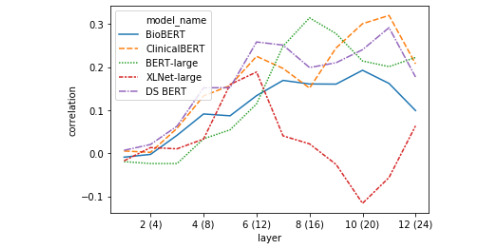
Correlation between the ground-truth model RDM and explanatory RDMs constructed using the absolute difference between each sentence’s mean-pooled token vector.

In carrying out the single-correlation RSA task, we found confirmation for some of the representational trends identified during the decoding task. Two of such trends are presented in Figures 8 and 10, which include the correlation of the model RDM with data RDMs built using classification tokens (Figure 8) and the absolute difference between average token vectors from the 2 sentences in a pair (Figure 10). As was previously shown in Figure 4, BERT-Large diverges drastically from the other models in how representative the classification token is of a sentence pair’s semantic similarity score around layers 12-16, while all other models generally generate progressively better [CLS] tokens throughout the network, with only slight loss in performance around the middle of the network. The performance of BERT-Large [CLS] representations on this task again reflects its final score, which was the lowest of the 5 models. We further analyzed the representational geometry of BERT-Large in our reweighting analysis later in the current section to better understand this observation. The confirmation of this considerable drop in decodability performance shows that this trend does not simply reflect the inability of the linear regression models to predict semantic information due to the small amount of data. Likewise, the correlation plot featured in Figure 10 presents more evidence for our previous finding that BERT-style models seem to represent across-sentence similarity by minimizing the average difference in token vectors. While these correlations are positive from layers 4 to 12, this signal is not as strong as would be indicated by the probing analysis, suggesting that this strategy may not be a primary heuristic. In any case, taken together, these 2 layer-wise correlation plots show that the probing task produces robust metrics of representational trends, and that probing and basic RSA are complementary approaches to the analysis of transformations in token vectors of deep transformer models.

##### Reweighted and Recombined RSA

**Figure 11:**
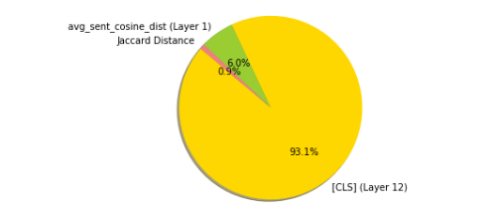
Proportion of weights learned for the best explanatory model (which used BioBERT representations and text features).

After performing the next stage in our RSA, reweighting and recombining a set of RDMs (using all layers using all representations, as well as the text features RDM) for each transformer to minimize the distance between the new RDM and the ground-truth representation, we found that the best choice of model was BioBERT. Figure 7 shows visual confirmation that much of the ground-truth dissimilarity patterning (Figure 6) has been reproduced by this explanatory model. This result was somewhat unexpected, given that this model did not perform best on the test set. This finding suggests that when generating sentence-pair vectors, it may in some cases be better to reweight and combine representations from runner-up models, rather than using the single best model. The weights learned for each RDM in the BioBERT model (Figure 11) show that the RDM is mostly made up of the final layer’s [CLS] token, although it has been reweighted using the cosine distance between the average token vector of the 2 sentences in a given pair and the Jaccard distance between the 2 sentences. We believe that beyond revealing how well each representation explains the ground-truth semantic similarity, this technique has promising potential for generating sentence embeddings for downstream tasks.

##### Layer-wise Reweighted RSA

By looking at the weights learned for each component of the layer-wise BERT explanatory model (Figure 9), we find that after layer 8, the weight associated with the average token representation drastically increases and this representation becomes dominant for the remaining layers, whereas the explanatory weight of the [CLS] token peaks at layer 8 before rapidly declining. We link this result to our finding that the worst linear probing and RSA correlations for BERT’s [CLS] tokens start to occur after layer 8 (Figures 4 and 8). This suggests that in middle to late layers, BERT-Large focuses on building better mean-pooled representations of the sentence pairs, an interpretation which is in line with the dramatic increase in correlation between BERT-Large’s representations and the ground-truth model when using the absolute difference between the average token vector of each sentence as the data RDM (Figure 10). This interpretation is also compatible with the increase in linear regression performance when using BERT-Large token vectors and taking the absolute difference between the average token vectors in each sentence as input (Figure 5).

### Limitations and Future Work

While we employed the use of cross-validation for our linear probing and NNLS RSA tasks, it should be noted that our test set of 412 sentence pairs represents a relatively small amount of data and as such it may be difficult to assess whether our results would generalize to more data-rich contexts. One potential method for partially mitigating this problem would be to cross-validate our results across the full set of 2054 sentence pairs, rather than restricting the analysis to the original test set from the clinical STS task. While this approach may lead to insights into the robustness of our interpretation, we consider it to be outside of the scope of this work as we aim to analyze the errors and representational strategies that both result from the inductive biases of transformer models and reflect biases learned from the task’s data. Restricting our analysis to the original 412 sentence-pair test set thus enables direct comparison with other models trained on the same data. Another issue with cross-validating across the whole data set is that we will always be limited to a relatively small amount of data for this task, as even testing on a slice of 50% of the total data would still only allow for 1027 sentence pairs for evaluation. It could also be insightful to carry out our analysis on models trained using larger general domain semantic similarity tasks that feature more sentence pairs. We again consider this line of research to be out of scope for this work.

In future work we wish to investigate to what extent we can directly use a layer’s token representations to automatically learn interpretable explanations that minimize the distance between a reweighted RDM and the ground-truth model RDM. We expect that incorporating our models’ attention weights will be essential at that level of analysis. Additionally, we wish to set alternative target RDMs to examine how we can recombine the token vectors in a sentence pair to best explain the model’s classification token, thereby further exploring the inner representational dynamics of fine-tuned transformer models.

### Conclusion

We tackled a recent clinical STS task using a variety of transformer models, including both those trained on general domain language and models that were further trained on clinical text. After achieving a high correlation between the predictions of a mean-pooled ensemble of these models and the test-set ground truth, we analyzed the error cases of our model both qualitatively and quantitatively, finding groups of semantically related sentences that are generally difficult for our transformers to model and identifying surface features of the sentence pair that significantly correlate with loss for particular ranges of the semantic similarity space. These findings suggest potential avenues for further improvement, for example, by augmenting our models to allow them to directly take traditional NLP textual features into account.

We then carried out 2 types of representational analyses, namely, linear decoding and RSA, to shed light on the heuristics on which these models have learned to rely. These approaches were shown to be complementary and revealed divergent representational strategies for predicting textual similarities between BERT-style models and XLNet. Furthermore, our search through the representational space for the best explanatory model of the ground-truth data suggests that a large amount of this information can be captured using a combination of a classification token and the cosine distance between sentence-pair representations in the first layer of a transformer model that did not produce the best predictions on the test set, suggesting interesting directions for research in model distillation and sentence embedding extraction.

## Abbreviations

BERT: bidirectional encoder representations from transformers
NLP: natural language processing
NNLS: non-negative least squares
RDM: representational dissimilarity matrix
RSA: representational similarity analysis
STS: semantic textual similarity
